# Assessment of refractive astigmatism and simulated therapeutic refractive surgery strategies in coma-like-aberrations-dominant corneal optics

**DOI:** 10.1186/s40662-016-0044-8

**Published:** 2016-05-12

**Authors:** Wen Zhou, Aleksandar Stojanovic, Tor Paaske Utheim

**Affiliations:** SynsLaser Kirurgi AS, Tromsø, Troms Norway; School of Ophthalmology and Optometry, Wenzhou Medical University, Wenzhou, China; Eye Department, University Hospital North Norway, Tromsø, Norway; Department of Ophthalmology, Vestre Viken Hospital Trust, Drammen, Norway; Department of Medical Biochemistry, Oslo University Hospital, Oslo, Norway; Department of Oral Biology, Faculty of Dentistry, University of Oslo, Oslo, Norway

**Keywords:** Coma, Higher-order-aberrations, Corneal irregular astigmatism, Topography-guided ablation

## Abstract

**Background:**

The aim of the study is to raise the awareness of the influence of coma-like higher-order aberrations (HOAs) on power and orientation of refractive astigmatism (RA) and to explore how to account for that influence in the planning of topography-guided refractive surgery in eyes with coma-like-aberrations-dominant corneal optics.

**Methods:**

Eleven eyes with coma-like-aberrations-dominant corneal optics and with low lenticular astigmatism (LA) were selected for astigmatism analysis and for treatment simulations with topography-guided custom ablation. Vector analysis was used to evaluate the contribution of coma-like corneal HOAs to RA. Two different strategies were used for simulated treatments aiming to regularize irregular corneal optics: With both strategies correction of anterior corneal surface irregularities (corneal HOAs) were intended. Correction of total corneal astigmatism (TCA) and RA was intended as well with strategies 1 and 2, respectively.

**Results:**

Axis of discrepant astigmatism (RA minus TCA minus LA) correlated strongly with axis of coma. Vertical coma influenced RA by canceling the effect of the with-the-rule astigmatism and increasing the effect of the against-the-rule astigmatism. After simulated correction of anterior corneal HOAs along with TCA and RA (strategies 1 and 2), only a small amount of anterior corneal astigmatism (ACA) and no TCA remained after strategy 1, while considerable amount of ACA and TCA remained after strategy 2.

**Conclusions:**

Coma-like corneal aberrations seem to contribute a considerable astigmatic component to RA in eyes with coma-like-aberrations dominant corneal optics. If topography-guided ablation is programmed to correct the corneal HOAs and RA, the astigmatic component caused by the coma-like corneal HOAs will be treated twice and will result in induced astigmatism. Disregarding RA and treating TCA along with the corneal HOAs is recommended instead.

## Background

**Fig. 1:**
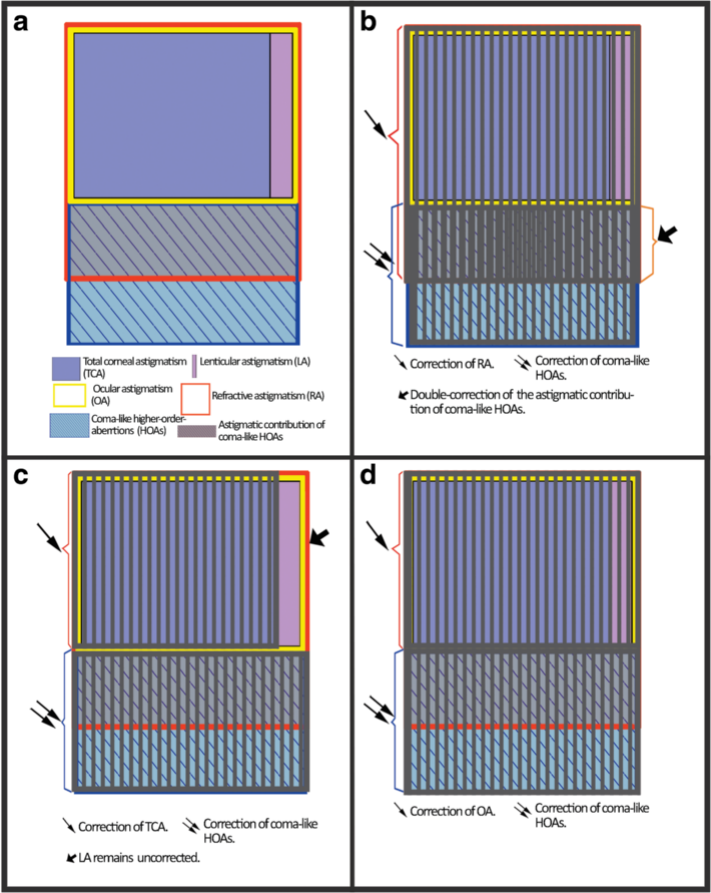
Schematic figure showing astigmatic components and effect of different treatment strategies. **a**. Different astigmatic components and coma-like HOAs. **b**. Simulated effect after strategy 2 (treating t along with coma-like HOAs), resulting in double correction of the astigmatic contribution of coma-like HOAs. **c**. Simulated effect after strategy 1 (treating total corneal astigmatism along with coma-like HOAs), resulting in uncorrected lenticular astigmatism. **d**. Simulated effect after correction of ocular astigmatism along with coma-like HOAs, resulting in full correction

Orthogonally asymmetric corneas have either different power amplitudes on the opposite sides of their astigmatic hemi-meridians or the hemi-meridians are not aligned along the same axis. This results in irregular optics, dominated by odd-order, higher-order aberrations (HOAs), most of which are coma and coma-like aberrations. It occurs in keratoconus [1, 2], cornel ectasia after laser in situ keratomileusis (LASIK) [3], decentered refractive surgery [4] and may also occur after any type of incisional corneal surgery, after pterygium-surgery [5], as well as after scarring due to corneal injuries or keratitis. In these conditions visual distortions and decrease in visual acuity occur irrespective of spherocylindrical error and its correction [6, 7]. Based on a computational model, it has been estimated that the refractive effect of a root-mean-square (RMS) HOA of 0.43 μm or greater is equivalent to at least 0.50 D of spherical error for a 5 mm pupil aperture [8, 9]. Wei and colleagues reported that the coma-like HOAs and trefoil contribute to (subjective) refractive astigmatism (RA) and that the amount of RA was directly influenced by the amount of horizontal coma and trefoil [10]. Alpins et al. also found that in the absence of lenticular astigmatism (LA), there was a difference between RA and the astigmatism measured by corneal topography [11]. This was ascribed to the contribution of the irregular astigmatism component i.e., the asymmetry of topographic hemi-meridians. During subjective refraction the patient is systematically presented with combinations of spherical and cylindrical lenses in the search for the one that forms the retinal images with least diffusion and distortion. Thus, the subjective refraction can neither determine the amount or type of HOAs nor their contribution to the resulting spherocylindrical refraction. This has a major impact in ablation planning in therapeutic refractive surgery cases with irregular astigmatism. If custom ablation, which treats HOAs is programmed to also treat RA, we would be treating the coma itself and its astigmatic effect within RA at the same time, resulting in a “double treatment” (Fig. 1a, b).

The goal of this study is to raise awareness of the contribution of coma-like HOAs to the amount and orientation of RA in eyes with coma-like-dominant corneal optics and to explore how to account for that contribution in the planning of therapeutic refractive surgery using topography-guided custom ablation.

## Methods

From the population of patients referred for therapeutic refractive surgery at the eye department of the University Hospital of North Norway, 11 eyes with coma-like-aberration-dominant optics due to keratoconus (eight eyes), LASIK flap complication (one eye), corneal scarring after photorefractive keratectomy (PRK) (one eye) and after keratitis (one eye) (Table 1) were selected for evaluation of contribution of their coma-like HOAs to their RA, as well as for the simulations of therapeutic topography-guided ablation aimed at regularization of corneal optics. Inclusion criteria were: 1) anterior corneal topography with orthogonal asymmetric power along any meridian ≥ 2 D, or misalignment of axis between principal hemi-meridians exceeding 10°; 2) vector difference between total corneal astigmatism (TCA) and subjective RA ≥ 1.5D; and 3) LA representing ≤ 30 % of the vector difference between total corneal and RA (Table 2).

**Table 1:** Demographic data for 11 cases

No.	Age	Eye	Gender	CDVA	Pupil (mm)^a^	Coma (μm)^b^	Diagnosis	Visual symptoms
1	33	os	Female	0.9	6.4	0.8	Keratoconus	Double images, starburst
2	33	os	Male	0.8	6.9	1.7	Keratoconus	Multiple images, haloes
3	44	od	Male	0.9	4.9	0.4	Keratoconus	Multiple images, starburst
4	44	os	Male	0.6	4.7	0.6	Keratoconus	Double images, starburst
5	49	os	Male	0.3	4.6	0.7	Keratoconus	Multiple images, starburst
6	27	od	Male	0.9	6.2	1.0	Keratoconus	Haloes, starburst
7	36	od	Male	1.0	6.0	0.7	scarring post PRK	Double images, starburst
8	60	os	Male	0.8	4.6	0.8	LASIK flap complication	Double images, starburst
9	20	os	Male	0.8	3.9	0.4	Keratoconus	Double images, starburst
10	24	od	Male	1.0	5.5	1.1	Keratoconus	Starburst
11	27	os	Male	1.0	4.8	0.5	Keratitis	Double images, starburst

*CDVA* = corrected distance visual acuity

^a^size of average of photopic and scotopic pupil

^b^RMS coma-like aberrations within individual pupil size, including primary coma from 3rd order and secondary coma from 5th order

**Table 2:** Deviation of different types of astigmatism and amount of coma-like aberration for 11 cases

No.	TCA	RA	IA	PCA	LA	LA/(RA-TCA)	DA	OA (TCA + LA)
1	−2.20/4	−0.5/95	−0.71/87	−0.16/113	−0.62/81	0.23	−2.16/98	−1.66/9
2	−3.40/176	−2.11/90	−1.45/95	−0.94/86	−0.63/109	0.11	−5.05/85	−2.99/172
3	−0.12/43	−2.95/95	−0.89/104	−0.17/80	−0.79/109	0.26	−2.29/92	−0.71/105
4	−0.37/170	−4.09/85	−1.29/88	−0.23/99	−1.08/86	0.24	−3.38/94	−0.72/89
5	−1.12/106	−6.36/110	−1.20/46	−0.51/40	−0.71/50	0.13	−5.65/114	−1.09/87
6	−1.94/121	−3.68/103	−0.75/96	−0.35/60	−0.67/110	0.28	−1.95/82	−2.58/118
7	−2.06/171	−1.51/46	−0.60/86	−0.30/142	−0.76/75	0.26	−2.22/64	−1.32/174
8	−0.43/31	−2.19/55	−0.27/39	−0.47/88	−0.57/12	0.30	−2.07/68	−0.95/20
9	−2.2/172	−0.50/80	−1.35/83	−0.57/84	−0.78/82	0.29	−1.92/81	−1.42/172
10	−1.14/142	−2.04/68	−1.00/90	−0.26/71	−0.81/96	0.24	−3.10/55	−1.59/127
11	−2.49/19	−0.96/20	−0.29/179	−0.08/174	−0.21/0.9	0.14	−1.71/106	−2.66/18

*TCA* = total corneal astigmatism, *RA* = refractive astigmatism, *IA* = internal astigmatism, *PCA* = posterior corneal astigmatism, *LA* = lenticular astigmatism, *DA* = discrepant astigmatism, *OA* = ocular astigmatism

### Astigmatism and coma-like aberration measurements

Since our main goal was to study the contribution of coma-like HOAs on RA in highly irregular corneas, measurements of corneal astigmatism and HOAs were obtained using different technologies to most objectively highlight the two components. We also chose the measurements that could be used in software simulations of topography-guided custom ablation, as it seems to be the most reasonable current approach in treatment of highly aberrated corneas [12, 13].

The magnitude and orientation of the TCA, measured by Scheimpflug topo/tomography (Precisio; iVIS Technology, Taranto, Italy) was calculated by ray tracing independent of the HOAs.

Internal astigmatism (IA), representing the astigmatism originating from the structures from the posterior corneal surface to the retina, was measured by OPD scan-II (NIDEK Co Ltd, Gamagori, Japan), which integrates automatic retinoscopy-based wavefront aberrometry and placido-based corneal topography. The difference between the total ocular astigmatism (which does not include the influence from HOAs) obtained by wavefront measurement and the anterior corneal astigmatism (ACA) obtained by corneal topography, gave the IA, consisting of the sum of the posterior corneal astigmatism (PCA) and the LA. The ACA was calculated using Snell’s law, using 1.3760 as the corneal refractive index and then translated to Zernicke polynomials after being adjusted for recentering from the corneal vertex to the line-of-sight, for the sake of compatibility with the ocular wavefront measurements. The IA differs from the ocular residual astigmatism as described by Alpins [14], which is calculated using manifest refraction and consequently includes the neural processing component as well.

PCA, measured by Precisio, was calculated using the equation (n´-n)/R, with 1.336(n') for refractive indexes for aqueous and 1.376(n) for cornea, and R as the posterior corneal curvature radius.

LA was calculated as the vector difference between IA and the PCA.

RA was obtained from non-cycloplegic manifest refraction. RA was first converted to cross-cylinder notation then transferred from spectacle plane to corneal plane using the vertex distance of 12 mm for direct comparison with the corneal astigmatism.

Discrepant astigmatism (DA) represents the discrepancy between RA and the sum of TCA and LA. Since the sum of TCA and LA represents the “pure” astigmatism excluding the effect of the coma-like HOAs. DA is normally negligible in eyes with normal optics but can become significant when coma-like HOAs refract as astigmatism. DA was calculated by vector analysis.

The orientation of astigmatism in the current article was presented as axis of the corrective cylinder (using minus cylinder for display in the tables to be accordant with usual practice, but using plus cylinder for double angle plot in the figures). RMS values of coma-like HOAs measured by wavefront aberrometry (OPD scan-II), were defined as square root of the sum of *c*_3_^1^, *c*_3_^− 1^, *c*_5_^1^ and *c*_5_^− 1^. Individual pupil size, which is an average of photopic and scotopic pupil, was used as the diameter at which astigmatism and coma-like HOAs for each case were analyzed.

**Fig. 2:**
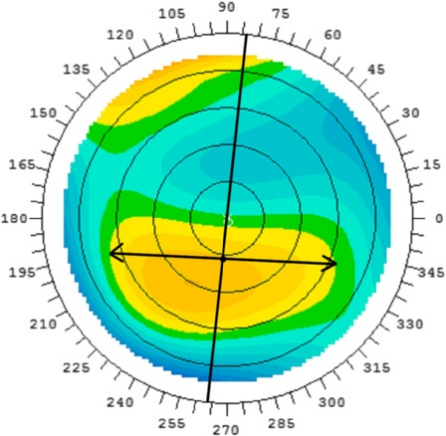
Measurement of axis of coma. Measurement of axis of coma on anterior corneal topography fitting by aconic surface

The axis of the corneal coma was defined as the axis passing through both corneal vertex and the midpoint of the specific elevated or depressed area on the anterior corneal elevation topography, using the aconic fitting (Fig. 2). Coma with axis oriented at 90° ± 30° was defined as vertical coma, and coma with axis oriented at 180° ± 30° was defined as horizontal coma. Coma with other orientations was defined as oblique coma.

### Simulations

Anterior corneal elevation maps obtained by Precisio were used as the basis for customized ablation design with Corneal Interactive Programmed Topographic Ablation software (CIPTA; Ligi, Taranto, Italy). CIPTA calculates simulated postoperative topography by subtracting the ablation map from the imported preoperative anterior topography map. The simulated postoperative topography shows the amount and the orientation of the postoperative corneal astigmatism (ACA, PCA and TCA).

The simulations comprised corneal vertex fitting with two different aconic surfaces of 6-mm in diameter, defined as the targeted surfaces for two different strategies: Strategy 1 aimed to correct TCA, along with the anterior corneal surface irregularities (the source of anterior corneal HOAs) i.e., using TCA and corneal topography as the basis for treatment of lower- and HOAs, respectively. Strategy 2 was aimed at correcting RA along with the anterior corneal surface irregularities i.e., using subjective refraction and corneal topography as the basis for treatment of lower- and higher-order corneal surface aberrations, respectively. In both cases the tissue between the existing anterior corneal surface and the targeted regular surface within the 6 mm would be ablated. The influence of subjective sphere and spherical-aberration-compensation were not specifically analyzed, as they were outside the scope of the simulations used for the current study and since the simulations were not used for the actual treatments.

### Vector analysis

Vector analysis was performed according to the method outlined by Jack T. Holladay [15, 16]: Each astigmatic value was converted to x and y Cartesian values (x = |Astigmatism| * Cos (2 * axis), y = |Astigmatism| * Sin (2 * axis)), and displayed using doubled- angle plots as positive cylinder notation.

The difference between RA and TCA (RA – TCA) and the difference between IA and PCA (IA – PCA) = LA, were calculated using vectors:

$$ \mathrm{R}\mathrm{A}\hbox{-} \mathrm{T}\mathrm{C}\mathrm{A}=\sqrt{{\left({x}_{RA}-{x}_{TCA}\right)}^2+{\left({y}_{RA}-{y}_{TCA}\right)}^2} $$

$$ \mathrm{L}\mathrm{A}=\mathrm{I}\mathrm{A}{\textstyle \hbox{-}}\mathrm{P}\mathrm{C}\mathrm{A}=\sqrt{{\left({x}_{IA}-{x}_{PCA}\right)}^2+{\left({y}_{IA}-{y}_{PCA}\right)}^2} $$

For most of the normal corneas, RA mainly originates from both TCA and LA i.e., RA-TCA = LA. However, for the corneas which are dominated by coma-like HOAs, RA would also be influenced by this irregularity [8–11]. This influence was defined as DA in the study, representing the discrepancy between RA and the total of TCA and LA. DA was calculated using the vectors as follows:

$$ \begin{array}{c}\mathrm{D}\mathrm{A}=\mathrm{R}\mathrm{A}{\textstyle \hbox{-}}\left(\mathrm{T}\mathrm{C}\mathrm{A}+\mathrm{L}\mathrm{A}\right)=\mathrm{R}\mathrm{A}{\textstyle \hbox{-}}\left(\mathrm{T}\mathrm{C}\mathrm{A}+\mathrm{I}\mathrm{A}{\textstyle \hbox{-}}\mathrm{P}\mathrm{C}\mathrm{A}\right)\\ {}=\sqrt{{\left({x}_{RA}-{x}_{TCA}-{x}_{IA}+{x}_{PCA}\right)}^2+{\left({y}_{RA}-{y}_{TCA}-{y}_{IA}+{y}_{PCA}\right)}^2}\end{array} $$

In the current study, we chose cases with relatively insignificant LA, manifested as LA representing ≤ 30 % of the vector difference between RA and TCA (Table 2). The calculation is shown as following:

$$ \mathrm{L}\mathrm{A}/\left(\mathrm{R}\mathrm{A}\hbox{-} \mathrm{T}\mathrm{C}\mathrm{A}\right)<30\% $$

The current study was approved by the Norwegian data protection authority and was granted exemption from the regional ethics committee (REK-NOR).

## Results

All eyes had above 0.3 μm RMS coma-like HOAs along with decreased corrected distance visual acuity (CDVA) and/or visual disturbances such as double/multiple images/contours, starburst, or haloes, not correctable by sphere and cylinder (Table 1).

**Fig. 3:**
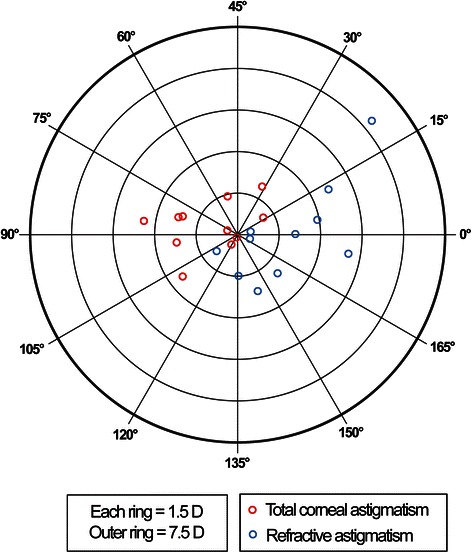
Total corneal astigmatism and refractive astigmatism. A double-angle, plus cylinder power plot of an 11-case set for total corneal astigmatism and refractive astigmatism

**Fig. 4:**
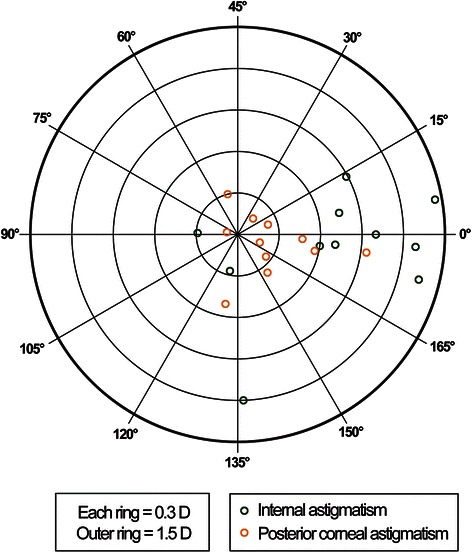
Internal astigmatism and posterior corneal astigmatism. A double-angle, plus cylinder power plot of an 11-case set for internal astigmatism and posterior corneal astigmatism

**Fig. 5:**
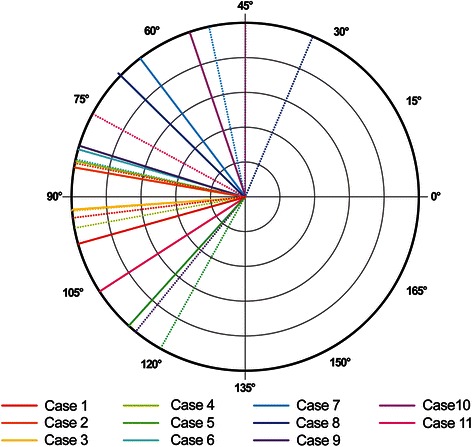
A double-angle plot of an 11-case set for orientation of coma-like aberrations and discrepant astigmatism

**Fig. 6:**
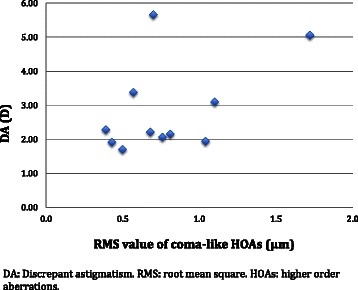
Scattergram showing the magnitude of coma-like HOAs and discrepant astigmatism. DA: Discrepant astigmatism. RMS: root mean square. HOAs: higher order aberrations

The distributions of TCA, RA, IA, and PCA for the 11 eyes are shown in Table 2 and Figs. 3–4. The axis of DA and axis of coma for each case is displayed in Table 4 and Fig. 5 and the amplitude of DA and RMS value of coma is shown in Fig. 6. Concerning the patients’ refractive error, only the astigmatism, including the refractive and all the objective measurements were analyzed and shown in Table 2.

**Fig. 7:**
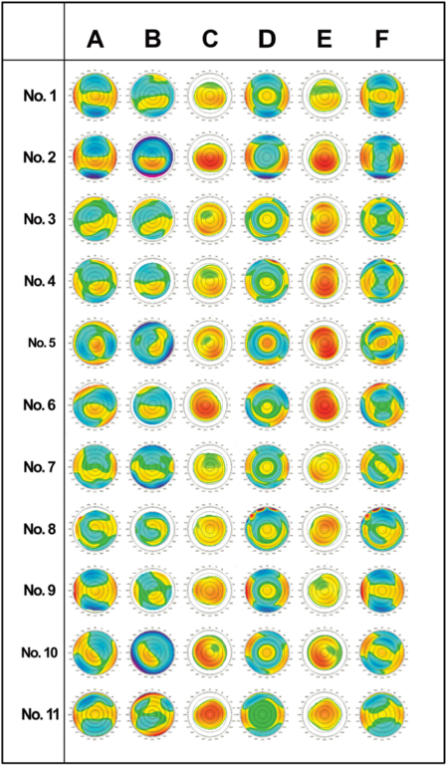
Simulated ablation maps and resultant postoperative anterior corneal elevation maps. Preoperative Scheimpflug based anterior corneal elevation maps fitting by sphere and aconic surface (column A and column B, respectively), simulated ablation maps based on total corneal astigmatism correction and resultant postoperative anterior corneal elevation maps fitting by sphere (column C and column D, respectively), simulated ablation maps based on refractive astigmatism correction and resultant postoperative anterior corneal elevation maps fitting by sphere (column E and column F, respectively)

**Fig. 8:**
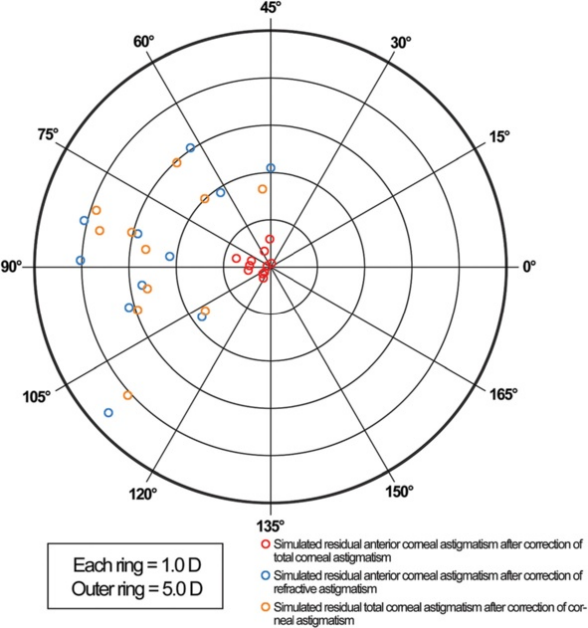
Postoperative astigmatism. A double-angle, plus cylinder power plot of an 11-case set for simulated postoperative astigmatism based on total corneal astigmatism correction and refractive astigmatism correction

The simulated postoperative ACA and TCA after using our two simulation strategies are presented in Table 3 and Figs. 7–8. After simulated correction of anterior corneal HOAs along with TCA and RA (strategies 1 and 2, respectively), only a small amount of ACA and no TCA remained after strategy 1, while considerable amounts of ACA and TCA remained after strategy 2.

**Table 3:** Simulated residual astigmatism for different strategies

	1	2	3	4	5	6	7	8	9	10	11
A.	−0.75@173	−0.59@136	−0.17@20	−0.36@146	−0.08@101	−0.22@20	−0.28@28	−0.45@178	−0.43@171	−0.48@4	−0.08@173
B.	−2.15@177	−4.03@179	−3.12@8	−4.07@173	−4.62@21	−2.75@4	−2.10@135	−1.90@152	−2.90@173	−3.04@152	−1.80@18
C.	−2.67@176	−3.70@174	−2.96@9	−3.88@171	−4.07@21	−2.65@5	−1.66@138	−2.01@157	−3.03@173	−2.97@156	−1.67@17

A. Simulated residual anterior corneal astigmatism after strategy 1 (correction of total corneal astigmatism)

B. Simulated residual anterior corneal astigmatism after strategy 2 (correction of refractive astigmatism)

C. Simulated residual total corneal astigmatism after strategy 2 (correction of refractive astigmatism)

## Discussion

RA is mostly correlated to corneal astigmatism due to the mere fact that the cornea contributes to more than 70 % of the total ocular refractive power [17]. Being the second most important refractive element, the crystalline lens may also contribute to RA. However, a significant discrepancy between RA and objectively measured ocular astigmatism is known to occur in conjunction with corneal pathologies resulting in HOAs-dominated optics as well. In the current study, we chose to analyze the cases that had coma-like-aberration-dominant optics with a difference between RA and TCA ≥ 1.5 D and with a relatively insignificant LA, in order to diminish the influence of LA as a source of DA.

Before the “wavefront” terminology was common, the term *irregular astigmatism* as introduced by Alpins [11] was used to describe corneal optics dominated by HOAs. Coma has been found to be the dominant HOA in asymmetric corneas [18], in decentered laser ablations [4], and in Keratoconus [1, 2, 9], where the corneal morphology changes are displaced from the optical center of the cornea, and form a coma-type wavefront aberration. Hence, the corneal morphological coma and the optical wavefront coma are highly associated especially in cases with no internal ocular coma. In the current study, we measured the location of coma and coma-like HOAs at the most asymmetric part of the corneal anterior elevation topography.

Manifest refraction, a common way to assess subjective sphere, astigmatism and visual acuity, is influenced by the amount, type and spatial distribution of corneal HOAs. It has been reported that spherical aberrations refract as hyperopia or myopia [8], while comas refract as astigmatism [10]. During phoropter testing the resultant subjectively refracted cylinder power and axis will be a vector sum of two components: One caused by the “real astigmatism” i.e., ocular astigmatism (the second order optical aberration with frequency of 2, originated from both cornea and lens) and the other caused by coma subjectively refracting as cylinder (Fig. 1a). The coma-like HOAs were calculated within the “individual pupil diameter” (a mean value between photopic and scotopic pupil) in order to match the situation of non-cycloplegic clinical examination under which the RA is measured and to estimate the influence of the coma-like HOAs on that measurement.

Our results show that the axis of the DA and the axis of coma correlated well (Table 4, Fig. 5). In three cases the difference in axes was slightly above 30° (cases 8, 9 and 11), but this difference may be ascribed to the influence of the concurrent trefoil present in those cases, as the trefoil was described to also influence the RA (Fig. 7) [10]. Table 4 shows that three cases had their vector sum of TCA and LA oriented with the rule (WTR), while their RA was against the rule (ATR), presumably due to the presence of vertical coma that influenced the RA. Also in the presence of vertical coma, four cases had their vector sum of TCA and LA oriented ATR. Their RA was also ATR, but of higher magnitude than the sum. In one case the vector sum of TCA and LA was oriented WTR, with RA also WTR but of lower magnitude than the sum. The effect of coma residing along an oblique axis with respect to the RA, as in the last three cases, was less obvious and would require a more complex analysis.

**Table 4:** Influence of coma on orientation of refractive astigmatism

	Axis of Coma	Axis of DA	TCL + LA	Coma	RA
1	94	98	WTR	Vertical	ATR
2	84	85	WTR	Vertical	ATR
3	97	92	ATR	Vertical	ATR↑
4	93	84	ATR	Vertical	ATR↑
5	119	114	ATR	Vertical	ATR↑
6	84	82	ATR	Vertical	ATR↑
7	51	64	WTR	Oblique	Oblique
8	34	68	WTR	Oblique	Oblique
9	115	81	WTR	Vertical	ATR
10	45	55	Oblique	Oblique	ATR
11	76	106	WTR	Vertical	WTR↓

*DA* = discrepant astigmatism; *TCA* = total corneal astigmatism, *LA* = lenticular astigmatism, *RA* = refractive astigmatism, *WTR* = with the rule, ATR = against the rule

↑: increased amplitude of astigmatism

↓: decreased amplitude of astigmatism

**Fig. 9:**
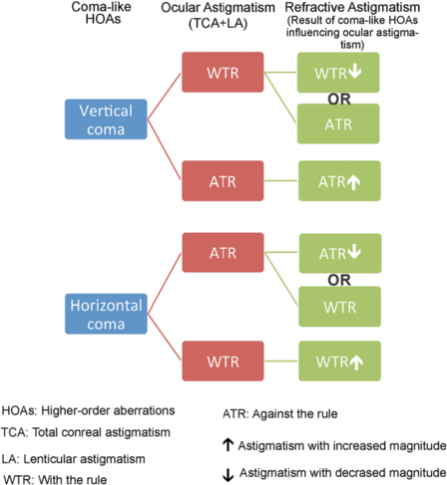
Schematic diagram showing the possible influence of coma on refractive astigmatism

The results above show that vertical coma is influencing the RA by canceling the effect of the WTR ocular astigmatism and increasing the effect of the ATR ocular astigmatism. In other words, vertical coma is refracted as ATR astigmatism. In the same manner, one could assume that the horizontal coma would be refracted as WTR astigmatism i.e., enhancing the effect of the WTR ocular astigmatism, and cancelling the effect of the ATR ocular astigmatism (Fig. 9).

The mechanism of how coma-like HOAs affect the subjective refraction has not been explored in the literature. For the 11 cases in the current study, there is no apparent correlation between the amplitude of coma-like HOAs and DA (Fig. 6). We would assume that the change in RA orientation is influenced by the location of the coma, but estimating the contribution from coma-like HOAs to the magnitude of RA seems to be more complex, involving pupil size during the manifest refraction examination, as well as the individual patient’s neural image processing.

In eyes with normal corneas, correcting refractive sphere and cylinder as measured by manifest refraction and using a non-customized laser ablation will lead to good postoperative visual outcomes in most cases. However, approaching the cases with aberrated corneal optics the same way will not include the treatment of HOAs and will most likely not result in improvement of the quality of the eye optics. If an ablation to treat regular astigmatism is performed based on the magnitude and axis orientation decided by subjective refraction i.e., not corresponding to the corneal astigmatism, new surface irregularities resulting in new HOAs will likely be induced.

### Why topography guided and not wavefront guided ablation?

When visual disturbances can be ascribed to corneal optical irregularities, it is more appropriate to use corneal topography as the source of data for custom ablation planning rather than the wavefront information from the entire optical system of the eye, since the former more closely represents the source of aberrations. Furthermore, the pupil-diameter limited operating principle of the ocular wavefront aberrometry may lead to unreliable results in corneas with distorted optics resulting from the pathologies residing outside the pupil, as in keratoconus and after pterygium, or incisional surgery. Meanwhile, precise (Scheimpflug-based) elevation topography portrays accurate corneal morphology thus resulting in more accurate corneal optics calculations. In addition, it provides the ability to map the posterior cornea and produce an accurate pachymetric and total corneal optics map. The primary measured elevation data is more accurate in determining the corneal morphology compared with wavefront aberrometry or even with Placido-technology-based curvature data, because the primary elevation measurements are not based on any assumed axis and therefore will not be influenced by displaced corneal apex [12, 13]. Depending on the calculation method and on how narrowly the corneal astigmatism is defined with respect to symmetry and alignment of its hemi-meridians, more or less of coma-like HOAs would be included in the value denominating astigmatism as measured by different instruments [19]. This may be an important reason for discrepancies in measurements of the astigmatism between different systems, rather than the differences in the raw data. The Precisio Scheimpflug topographer system used in the current study defines TCA (anterior plus posterior) by ray tracing and wavefront error estimation and is supposed to provide astigmatism measurements not influenced by coexisting HOAs.

### Why total corneal astigmatism and not anterior corneal astigmatism?

Total corneal power has traditionally been calculated on the basis of the anterior corneal curvature, using the keratometric refractive index of 1.3375, to compensate for the effect of the posterior surface. Neglecting the real influence of the posterior corneal surface in vision-correction planning may not result in errors if the profiles of anterior and posterior surfaces follow each other. However, if there is a discrepancy between the two, the influence of the posterior surface on the total corneal refraction can be significant [20, 21]. In keratoconus or irregular astigmatism secondary to incisional refractive surgery, major morphological changes originate from the posterior surface and are optically balanced by less pronounced changes of similar profile on the anterior surface due to epithelial remodeling [22]. In such cases, regularization of the anterior surface via topography-guided ablation may break that balance and lead to the manifestation of the latent refractive errors originating from the posterior surface. This has been shown in some cases in which significantly increased ocular HOAs and lower visual performance were seen in keratoconic eyes upon correction with rigid gas-permeable lenses [23, 24]. The simulation in the current study based on strategy 1 recognizes this issue and uses the TCA as the basis for corneal optical regularization. It showed that simulated ablation aimed at correction of TCA and corneal HOAs results in a regularized anterior corneal surface with a low level residual ACA compensating the PCA (Table 3), while its ablation map reflects correction of the morphological coma and TCA. As expected, a better symmetry of the simulated postoperative anterior corneal topography using strategy 1 was also achieved compared to the symmetry of the simulated postoperative topography after the ablation based on strategy 2 (aimed at correction of RA and corneal HOAs) (Fig. 7).

### Why is the topography-guided strategy where TCA (along with the corneal HOAs) is treated preferable to treating RA (along with the corneal HOAs)?

RA in the presence of coma-like HOAs and low LA represents a vector sum of HOAs refracting as astigmatism and TCA (Fig. 1a). When the corneal HOAs and the TCA are treated, all the sources of RA, except for LA, are addressed (Fig. 1c). However, when corneal HOAs and RA are treated, then the corneal coma itself, as a part of the corneal HOAs and its effect on RA are both treated. This amounts to a double treatment of the subjective astigmatic component i.e., removal of its cause and at the same time as the treatment of its effect (Fig. 1b).

### Why not use the total ocular astigmatism acquired by aberrometry?

Using total ocular astigmatism acquired by aberrometry would clearly have been the most elegant choice to determine the magnitude and orientation of the astigmatism to be treated (Fig. 1d), but the quality of the aberrometry data in highly aberrated corneas is often insufficient and cannot be used in topography-guided ablation without compatibility issues.

### Shortcomings of the study

The main shortcoming of the study is a possible error due to combination of the data acquired by two separate instruments using three different types of technologies (Scheimpflug- and Placido-based topography along with OPD-based wavefront aberrometry) for calculation of the LA. In addition to the registration error that may occur between any two separate examinations, one must take into consideration the potential error due to compatibility issues. Ideally, one instrument, using one type of technology, should be used for the measurement of the LA.

A support for the outlined treatment strategy by real results would have been desirable, as the real outcomes would have been influenced by the healing and biomechanical responses, as well as epithelial remodeling, all of which cannot be accounted for in the current simulations. However, the eight keratoconus cases were not candidates for refractive surgery, while the other three treated cases were patients coming from abroad and therefore not being available to undertake their follow-up examinations under the conditions needed for a meaningful analysis. Finally there were too few cases for a global statistical analysis.

## Conclusions

To our knowledge, this is the first study looking closer at the influence of coma-like HOAs on RA, as well as on the implications this issue brings to custom ablation planning in eyes with coma dominated optics. Our study shows that coma-like HOAs may have substantial influence on RA, depending on its amount and orientation with respect to TCA. Topography-guided custom ablation which aims to correct corneal HOAs along with TCA independent on RA appears to be the preferred treatment strategy in dealing with this issue, except in cases with significant LA.
